# Filoviruses: Innate Immunity, Inflammatory Cell Death, and Cytokines

**DOI:** 10.3390/pathogens11121400

**Published:** 2022-11-23

**Authors:** Jianlin Lu, Jessica M. Gullett, Thirumala-Devi Kanneganti

**Affiliations:** Department of Immunology, St. Jude Children’s Research Hospital, Memphis, TN 38105, USA

**Keywords:** innate immunity, inflammation, pattern recognition receptors, filovirus, ebolavirus, marburgvirus, RNA virus, host–pathogen interactions, cell death, pyroptosis, apoptosis, necroptosis, PANoptosis, PANoptosome, inflammasome, caspase, TLRs, RIG-I, RLRs, NLRs, ALRs, CLRs, interferon

## Abstract

Filoviruses are a group of single-stranded negative sense RNA viruses. The most well-known filoviruses that affect humans are ebolaviruses and marburgviruses. During infection, they can cause life-threatening symptoms such as inflammation, tissue damage, and hemorrhagic fever, with case fatality rates as high as 90%. The innate immune system is the first line of defense against pathogenic insults such as filoviruses. Pattern recognition receptors (PRRs), including toll-like receptors, retinoic acid-inducible gene-I-like receptors, C-type lectin receptors, AIM2-like receptors, and NOD-like receptors, detect pathogens and activate downstream signaling to induce the production of proinflammatory cytokines and interferons, alert the surrounding cells to the threat, and clear infected and damaged cells through innate immune cell death. However, filoviruses can modulate the host inflammatory response and innate immune cell death, causing an aberrant immune reaction. Here, we discuss how the innate immune system senses invading filoviruses and how these deadly pathogens interfere with the immune response. Furthermore, we highlight the experimental difficulties of studying filoviruses as well as the current state of filovirus-targeting therapeutics.

## 1. Introduction

Innate immunity provides the first line of defense to sense and respond to pathogens, which is crucial for host survival. As a result, there is competition between the host and pathogen, providing evolutionary pressure to select optimal host innate immune responses and pathogen evasion strategies. The molecular mechanisms underlying innate immunity and pathogen evasion strategies have proven to be key targets to inform cutting-edge therapeutic strategies. While many advances have been made in understanding host–pathogen interactions and immune responses, one class of viruses has proven difficult to study: the filoviruses.

Filoviruses are a group of single-stranded negative sense RNA viruses within the family *Filoviridae*. There are five established genera (*Ebolavirus*, *Marburgvirus*, *Cuevavirus*, *Striavirus*, *Thamnovirus*) and one proposed genus (*Dianlovirus*) [1]. The most well-known human pathogenic filoviruses are ebolaviruses and marburgviruses. These can cause high fever, systemic shock, elevated liver enzymes, fatigue, myalgia, coagulation defects, and death in humans and nonhuman primates [2]. Mental confusion and changes in personality also have been reported [3]. *Marburgvirus*, composed of both Marburg virus and Ravn virus, was first found in 1967 in Germany and Yugoslavia, when people handling monkey tissue succumbed to hemorrhagic fever and death [4,5,6,7]. Nine years later in the Democratic Republic of Congo (formerly known as Zaire) and in Sudan, the first recorded outbreaks of ebolavirus occurred, which were caused by two distinct viral species, *Zaire ebolavirus* and *Sudan ebolavirus* [8]. In addition to these species, *Taï Forest ebolavirus*, *Bundibugyo ebolavirus*, *Bombali ebolavirus*, and *Reston ebolavirus* have also subsequently been discovered [1]. 

**Figure 1:**
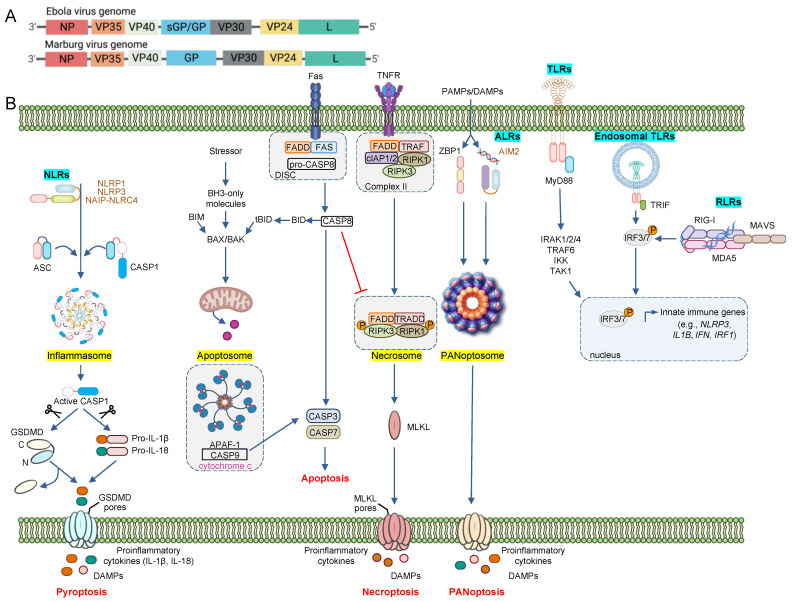
**Filovirus genetic organization and the innate immune sensors and their downstream cell death signaling pathways**. (**A**) Schematic illustration of Ebola virus and Marburg virus genomes. NP, nucleoprotein; VP, viral protein; GP, glycoprotein; sGP, secreted glycoprotein; L, RNA-dependent RNA polymerase. (**B**) Pathogenic insults such as filovirus infection can activate PRRs such as TLRs, RLRs, CLRs, NLRs, and ALRs. Activation of PRRs, as well as intracellular stressors, induces inflammation and innate immune cell death pathways such as pyroptosis, apoptosis, necroptosis, and PANoptosis. ALR, absent in melanoma 2 (AIM2)-like receptor; APAF-1, apoptotic protease activating factor 1; ASC, apoptosis-associated speck-like protein containing a CARD; CARD, caspase activation and recruitment domain; CASP, caspase; cIAP, cellular inhibitor of apoptosis protein; CLR, C-type lectin receptor; DAMPs, damage-associated molecular patterns; DISC, death-inducing signaling complex; FADD, fas-associated death domain; GSDMD, gasdermin D; IKK, inhibition of nuclear factor-κB kinase; IRAK, interleukin-1 receptor-associated kinase; IRF, interferon regulatory factor; MAVS, mitochondrial antiviral signaling protein; MDA5, melanoma differentiation-associated protein 5; MLKL, mixed lineage kinase domain-like pseudokinase; NLR, NOD-like receptor; PAMPs, pathogen-associated molecular patterns; PRR, pattern recognition receptor; RIG-I, retinoic acid-inducible gene-I, RLR, RIG-I-like receptor; RIPK, receptor-interacting serine/threonine-protein kinase; TAK1, transforming growth factor-beta-activated kinase 1; TLR, toll-like receptor; TNFR, tumor necrosis factor receptor; TRAF, tumor necrosis factor receptor-associated factor; TRADD, tumor necrosis factor receptor 1-associated death domain protein; TRIF, TIR-domain-containing adapter-inducing interferon-β; ZBP1, Z-DNA-binding protein 1. Figure created with Biorender.

Structurally, the filovirus virion is approximately 80 nm in width and variable in length; highly infective virions are approximately 790 nm for marburgviruses and 970 nm for ebolaviruses [8]. Each molecule of single-stranded negative sense RNA is enveloped by a lipid membrane obtained from the host plasma membrane during viral budding [9]. Filoviruses are filamentous in nature and contain seven genes encoding the following proteins: nucleoprotein (NP), RNA-dependent RNA polymerase (L), viral protein 24 (VP24), viral protein 30 (VP30), viral protein 35 (VP35), viral protein 40 (VP40), and surface glycoprotein (GP). Ebolaviruses also produce secreted glycoprotein (sGP) (Figure 1A) [8].

To infect, filovirus virions attach to host cells using host attachment factors, such as the asialoglycoprotein receptor (ASGP-R), C-type lectins, T-cell immunoglobulin, mucin domain 1 (TIM-1), and Niemann-Pick C1 (NPC-1) [10,11,12,13,14,15,16,17,18,19]. Additional factors have also been reported to regulate cell entry, including homotypic fusion and the vacuole protein-sorting (HOPS) multisubunit tethering complex, the cysteine proteases cathepsin L and cathepsin B, and signaling molecules, including acid sphingomyelinase and phosphoinositide-3 kinase [19,20,21,22,23,24,25]. Viral attachment allows the viral membrane to fuse with the host endolysosomal membrane and release viral nucleocapsids into the cytoplasm to begin viral replication and transcription [26,27,28]. Ebola GP is important in both receptor binding and membrane fusion. The immune system produces antibodies against Ebola GP, which, rather than inhibiting the virus, increases Ebola virus infectivity via antibody-dependent enhancement of infection (ADE) [29]. ADE also occurs through the complement component, C1q [30]. 

Filoviruses are highly contagious, and some marburgviruses and ebolaviruses have case fatality rates as high as 90% [31,32]. In addition to the high case fatality rates, filoviruses have multiple effects on the innate immune response. In the early stage of filovirus infection (days 1–4), when the virus first enters the human host through mucosal surfaces, innate immune cells including monocytes/macrophages and dendritic cells (DCs) are the first to become infected [33,34]. Common symptoms are fever, headache, body aches, and fatigue [35]. Viral proteins disrupt the host innate immune signaling pathways to block antiviral signaling, allowing robust viral replication in these cells [36]. As viral replication ensues, the early organ phase emerges (days 5–13) accompanied by gastrointestinal issues and declining health. In response to this stage of infection, the immune cells produce high levels of proinflammatory cytokines and chemokines [18,36,37,38,39]. Multiple studies show that those with severe filovirus infections have higher serum levels of proinflammatory biomarkers such as such as IFN-γ, IL-2, IL-8, IL-10, TNF-α, and IFN-α at various points in the infection compared to those with less severe disease [40,41,42,43]. This increase in proinflammatory cytokines recruits additional macrophages and DCs. These recruited cells also become infected and spread the virus to other tissues as they circulate. Ultimately, this spread leads to the late organ/convalescence phase where systemic vascular leakage, hemorrhage, organ failure, and disruption of adaptive immune responses occur [18,35]. 

In this review, we will discuss how the innate immune system senses and responds to pathogens and how filoviruses molecularly disrupt the immune system, resulting in a sub-optimal initial immune response that allows for rapid viral replication that is accompanied by inflammation, pathogenesis, and death in later stages of infection. Filoviruses have been shown to interact with a variety of innate immune cells [36], and filovirus-derived virus-like particles do activate natural killer cells [44,45,46], but here we limit our discussion to macrophages and DCs for brevity, as they are the initial innate immune cells infected by filoviruses and also serve as the major cellular target and the preferred viral replication site [33,34,35,47]. We will also discuss limitations surrounding the study of filoviruses and the current state of filovirus-targeting therapeutics and prevention strategies. Due to their high fatality rate and ability to modulate innate immune activation, improved understanding of the interplay between filoviruses and innate immunity will be critical for finding treatment strategies for these infections. 

## 2. Sensors of the Innate Immune System: The First Line of Defense against Pathogens 

To defend against pathogens and cellular perturbations, the innate immune system has evolved the ability to sense and respond to pathogen-associated molecular patterns (PAMPs) and danger- or damage-associated molecular patterns (DAMPs). PAMPs and DAMPs are sensed through germline-encoded pattern recognition receptors (PRRs), including membrane-bound toll-like receptors (TLRs) and C-type lectin receptors (CLRs), as well as cytoplasmic retinoic acid-inducible gene-I (RIG-I)-like receptors (RLRs), NOD-like receptors (NLRs), and absent in melanoma 2 (AIM2)-like receptors (ALRs) [48,49]. Once activated, PRRs drive innate immune responses such as the production of proinflammatory cytokines and interferons (IFNs) and the induction of innate immune cell death to clear the infected or damaged cell (Figure 1B) [48,49]. These processes are crucial for the activation of a broader immune response. 

The collective activation of multiple PRRs upon infection allows for a sensitive and finely tuned immune response that can provide optimal protection to the host. Below, we describe how the different classes of PRRs activate an innate immune response and what is known about each type of PRR as it pertains to filovirus pathogenesis. Though some information is known about specific PRRs involved in sensing and responding to filoviruses, additional studies are required to fully characterize how the innate immune system responds to these pathogens. Here, we will limit our discussion to the sensors and regulatory processes in mammalian systems (human, nonhuman primates, and mouse models that use mouse-adapted strains of filoviruses). There are many non-human reservoirs for filoviruses, such as bats, but the innate immune sensing in these animals differs from that in humans and nonhuman primates and has been reviewed elsewhere [50].

### 2.1. Toll-like Receptors

TLRs are a class of well-characterized PRRs highly expressed in innate immune cells such as macrophages, DCs, and mast cells; they can also be expressed in non-immune cell types, including epithelial cells and fibroblasts [51]. Ten TLRs exist in humans, and they are present in the plasma membrane (TLR1, -2, -4, -5, -6, -10) and on endolysosomal membranes (TLR3, -4, -7, -8, -9). Structurally, TLRs consist of an extracellular N-terminal ectodomain that recognizes ligands, a transmembrane domain, and a cytoplasmic C-terminal domain involved in signal transduction [52]. TLRs respond to a variety of stimuli such as bacterial lipopolysaccharide (TLR4), microbial components such as lipoteichoic acid, lipoproteins, and flagellin (TLR1, -2, -4, -5, and -6), and single- and double-stranded RNA and DNA (TLR-3, -7, -8, -9) [51]. All TLRs except TLR3 use the cytosolic Toll/IL-1 receptor (TIR) domain-containing protein adaptor, MyD88, to propagate signaling, though TLRs can also use additional adaptors such as TIRAP (TLR2, -4), TRIF (TLR3, -4), and TRAM (TLR4) [53]. MyD88, along with IL-1 receptor-associated kinase 1 (IRAK1), IRAK2, IRAK4, TNFR-associated factor 6 (TRAF6), transforming growth factor-β activated kinase 1 (TAK1), and the IKK complex, activate transcription factors (e.g., NF-κB, AP-1, IRF3, IRF7). Additional transcription factors can also be activated through TANK binding kinase 1 (TBK1). During infection, TRIF, as well as MAVS and STING, which will be discussed in a later section, promote the activation of TBK1 to facilitate phosphorylation and activation of IRF3. Subsequent nuclear translocation of these transcription factors promotes gene expression of the target innate immune genes [54]. 

TLR activation can also induce the production of cytokines, secreted proteins that allow cellular communication in an autocrine, paracrine, or endocrine manner [55]. Cytokines can be proinflammatory, such as IL-1β, IL-6, and TNF-α, and alert other immune cells to the infection [55], or they can be anti-inflammatory. While proinflammatory cytokines are important to induce an antiviral state, overproduction can result in a localized inflammatory reaction and a positive feedback loop between cytokine production and inflammatory cell death, leading to systemic inflammation, cytokine storm, multiple organ failure, and death [56]. Furthermore, the adaptive immune system also has critical roles in perpetuating cytokine storms during infections such as the Ebola virus [57]. For these reasons, proinflammatory cytokines such as IL-1β and TNF-α have been evaluated as therapeutic targets, as their inhibition can mitigate the hyperinflammatory state of the host [58,59,60]. 

TLRs have been shown to play a role in ebolavirus pathogenesis. In vitro analyses showed that TLR4 can respond to glycosylated Ebola GP, suggesting that the glycosylation pattern of this viral protein could act as a DAMP recognized by TLR4 [61]. Overall, the binding of GP to TLR4 leads to activation of the innate immune system and also influences immune cell differentiation [62,63]. The use of a TLR4 antagonist throughout all stages of infection promotes survival in mice infected with either mouse-adapted Ebola virus or Marburg virus. Ebola virus-infected mice treated with the TLR4 antagonist displayed a reduction in serum levels of inflammatory cytokines such as TNF-α, IL-6, IL-9, IL-10, and IL-13, as well as multiple chemokines [62]. Furthermore, Reston virus, which has only been shown to infect nonhuman primates, cannot stimulate TLR4, which suggests that this TLR is particularly important for filovirus pathogenicity in humans [64]. Additionally, in a study using Ebola virus-like particles, the TLR adaptors MyD88 and TRIF were both important for inciting a host innate immune response, thereby giving some insight as to how filovirus infections are sensed by TLRs [65]. While other TLRs have been implicated in a variety of infections [55], not much is known about TLRs, aside from TLR4, in the context of filovirus infections. 

### 2.2. RIG-I-like Receptors and IFN Signaling

**Figure 2:**
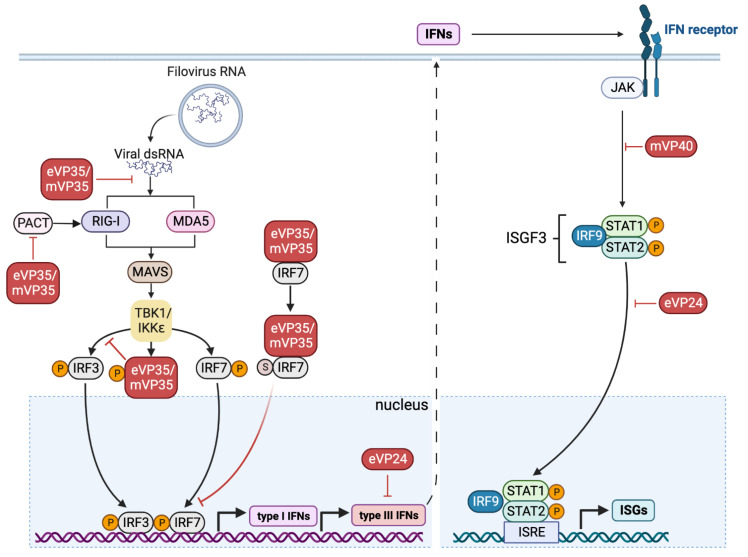
**Illustration of filovirus modulation of the RLR signaling pathway.** A typical RLR-mediated immune response to viral RNA activates RIG-I and MDA5. Downstream of RLR activation, IRF3 and IRF7 are phosphorylated through a TBK1/IKK-dependent pathway. The phosphorylation of these transcription factors leads to the production of IFNs, which stimulate the JAK/STAT pathway and allow for the transcription of ISGs. Filoviruses can disrupt this innate immune pathway largely through Ebola virus VP35 (eVP35) and Marburg virus VP35 (mVP35). Both forms of VP35 can directly bind viral RNA, which prevents its recognition by RLR sensors, disrupts RIG-I activation by targeting PACT, acts as an alternate substrate for IKKɛ and TBK1, and prevents the phosphorylation of IRF3. In addition, VP35 can cause sumoylation of IRF7 to inhibit its transcription of type I IFNs. eVP24 inhibits type III IFN secretion through an importin-α-dependent nuclear mechanism and inhibits the translocation of phosphorylated STAT1. mVP40 inhibits JAK1-mediated signaling. IFN, interferon; ISG, IFN-stimulated gene; IKKɛ, inhibitor of nuclear factor kappa-B kinase subunit epsilon; IRF, interferon regulatory factor; ISGF3, ISG, interferon stimulated gene factor 3; ISRE, interferon-sensitive response element; JAK, tyrosine-protein kinase; MAVS, mitochondrial antiviral-signaling protein; MDA5, melanoma differentiation-associated protein 5; PACT, protein kinase R (PKR) activator; RIG-I, retinoic acid-inducible gene-I; STAT, signal transducer and activator of transcription; TBK1, TANK-binding kinase 1; VP24, viral protein 24; VP35, viral protein 35; VP40, viral protein 40. Figure created with Biorender.

Most human cell types express RLRs in the cytosol, though some RLRs have been visualized in the nucleus [66]. Three human RLRs have been identified to date: RIG-I, melanoma differentiation-associated factor 5 (MDA5), and laboratory of genetics and physiology 2 (LGP2) [67]. Though some structural differences exist, RIG-I and MDA5 contain two N-terminal caspase activation and recruitment domains (CARDs), two DExD/H box RNA helicase domains, and a C-terminal domain (CTD); by contrast, LGP2 lacks a CARD [68,69,70]. Both RIG-I and MDA5 detect viral RNA and produce IFNs in response to infection (Figure 2). RIG-I preferentially recognizes short dsRNA and ssRNA, whereas MDA5 recognizes long dsRNA [71]; furthermore, RIG-I responds efficiently to negative strand viruses, and MDA5 to positive strand viruses [71]. Once activated by viral RNA, RLRs associate with mitochondrial antiviral-signaling protein (MAVS or IPS1) through a CARD–CARD interaction [72]. Interaction with MAVS targets RLRs to the mitochondrial membrane to form a signaling complex that drives type I and type III IFN production and IFN-stimulated gene (ISG) expression [73,74]. Post-translational modifications of RIG-I through E3 ligases and ubiquitinases, such as TRIM25 (tripartite-motif family member 25), Riplet, RNF135, TRIM4, and MEX3C, can alter the RIG-I–MAVS interaction and affect the strength of the RLR-mediated antiviral response. For example, TRIM25 promotes RIG-I oligomerization and interaction with MAVS to induce antiviral gene expression [75]. RLRs also have additional layers of regulation; though RLRs such as RIG-I are expressed at basal levels, IFN stimulation during infection greatly increases their expression [76].

The IFN signaling pathway, and particularly the production of type I IFNs (IFN-α, IFN-β), is key in establishing an antiviral state in the cell. Virus-induced TLR and RIG-I/MDA5 activation results in the phosphorylation and activation of IRF3, IRF7, NF-κB, and AP-1; these transcription factors translocate to the nucleus and promote the transcription of IFNs (Figure 2) [77]. Type I IFNs are then secreted by the cell and bind to their cognate cell surface IFN receptor (IFNAR1/2), which is ubiquitously expressed by most cells [77]. This binding event causes the phosphorylation of JAK1 and activation of the JAK/STAT pathway. Ultimately, IRF9 is recruited to the JAK/STAT complex to form ISG factor 3 (ISGF3), which translocates to the nucleus and binds ISG DNA sequences [78]. The subsequent upregulation of ISGs promotes an antiviral state by disrupting the viral lifecycle, blocking viral entry, degrading viral proteins, and blocking nuclear transport, among other functions [78]. The type I IFN response not only limits pathogenic spread but also can modulate the host response, and therefore control cytokine production as needed [18]. Viruses can also induce RIG-I-mediated type III IFN (IFN-λ1) production, especially in epithelial cells [79]. Though activated by separate receptors, type I and type III IFNs can be activated by the same transcription factors and share similar downstream signaling pathways (JAK/STAT activation) [80,81]. 

There are many ways in which filoviruses interfere with RLR-mediated signaling (Figure 2, Table 1). Much of what we know about RLR interference by filoviruses has been tested in vitro and, therefore, the timing of these effects on early versus late viral infection requires further study. Canonically, downstream of RIG-I or MDA5 activation, TBK1/IKKɛ-mediated IRF3 and IRF7 transcription drive host IFN production to repress the virus. However, many studies using filovirus proteins have shown that filoviruses have evolved mechanisms to escape these antiviral responses and avoid host cell death [37,82,83]. VP35, found in both Ebola viruses (eVP35) and Marburg viruses (mVP35), can bind viral dsRNA and prevent recognition of the virus by RIG-I and MDA5 [84,85]. VP35 can also inhibit RIG-I signaling by targeting PACT (protein kinase R activator), which is essential for RIG-I activation [86,87]. VP35 is crucial to the pathogenesis of filoviruses, as it prevents the phosphorylation of IRF3 and halts the host IFN response. VP35 can act as a decoy substrate for IKKɛ and TBK1; the resulting interaction leads to phosphorylation of VP35 instead of IRF3. As for IRF7, its interaction with VP35 leads to IRF7 sumoylation, which hinders transcription [88]. By blocking IRF3- and IRF7-mediated IFN production, VP35 prevents activation of the JAK/STAT pathway, which would normally elicit an antiviral state in the cell. For cells that do activate the JAK/STAT pathway, filoviruses can also target molecules in this pathway; eVP24 blocks the nuclear translocation of phosphorylated STAT1 [89,90,91], and mVP40 can inhibit JAK1-mediated signaling [92]. In addition, eVP24 has also been shown to inhibit IFN-λ1 production through an importin α-dependent mechanism. This inhibition does not interfere with IRF3 phosphorylation and is suggested to take place in the nucleus [79,93].

RIG-I and MAVS are the most well-studied RLRs, and PRRs in general, for their response to filoviruses. Less is known about the role of LGP2 in filovirus infection. Recent data have shown that LGP2 may play a role in regulating RIG-I and MDA5 [68,95,96,97,98], but it is unknown whether filovirus infection may influence this regulation. 

### 2.3. C-Type Lectin Receptors 

CLRs sense carbohydrates on molecular surfaces. CLRs can be categorized into three groups: type I transmembrane CLRs, which have an extracellular N-terminus and several carbohydrate recognition domains (CRDs); type II transmembrane CLRs, which have an intracellular N-terminus with one CRD; and soluble CLRs [99]. For type I and type II transmembrane CLRs, the CRDs determine glycan binding specificity; most human CLRs sense the amino acid motif Glu-Pro-Asn. While glycan sensing makes CLRs critical for host detection of fungi, CLRs have also been implicated in filovirus entry into host cells. Glycoproteins, such as those produced by filoviruses, are decorated with glycans, mostly *N*-linked glycans, which can be recognized by CLRs to induce endocytosis [100], allowing the virus to enter the cell. 

Dendritic cell-specific intercellular adhesion molecule grabbing nonintegrin (DC-SIGN) is a C-type lectin that, along with DC-SIGN related protein (DC-SIGNR), may serve as an attachment factor that enhances the spread of filoviruses [101]. DC-SIGN is expressed on DCs and macrophages, while DC-SIGNR is expressed on liver endothelial cells and lymph node sinusoids [102]. Ebola GP, which is glycosylated, can bind to these molecules to assist in viral entry and propagate infection. Efficient viral entry is thought to depend on the glycosylation of GP, as the presence of a high proportion of mannose *N*-glycans enhances viral infection the most [103]. However, it is difficult to fully understand the role of this glycosylation and DC-SIGN–mediated filovirus entry, as filoviruses differentially glycosylate their GPs, and variation occurs both between and within a single isolate [103]. 

Two other CLRs, ASGP-R and human macrophage galactose-type C-type lectin (hMGL), have been implicated in host–filovirus interactions. ASGP-R binds Marburg virus in hepatocytes, whereas hMGL, a type II transmembrane CLR, is unique in its ability to specifically bind galactose and *N*-acetylgalactosamine and increases Ebola virus infectivity [11]. However, the increased infectivity is dependent on which ebolavirus or marburgvirus is used [11], and it is likely that other factors are involved and influence CLR-mediated filovirus entry into host cells. Currently, it is unknown if or how CLRs are involved in sensing filoviruses to drive immune activation. 

### 2.4. NOD-like Receptors

NLRs respond to a variety of PAMPs and DAMPs. These highly conserved PRRs are structurally characterized by an N-terminal signaling domain, an oligomerization domain (NACHT), and a C-terminus with Leu-rich repeats (LRRs) [104]. Four subfamilies of NLRs exist, and they are differentiated through their N-terminal domain: NLRA or Class II transactivator (CIITA), NLRBs or NLR family apoptosis inhibitory proteins (NAIPs), NLRCs, and NLRPs. Some NLRs form multiprotein complexes called inflammasomes, which can activate caspase-1 to cleave its proteolytic substrates, including proinflammatory cytokines (IL-1β and IL-18) and the pore-forming molecule gasdermin D (GSDMD) to induce membrane pore formation and cell death [105]. One of the most well-studied inflammasomes is the NLRP3 inflammasome, and subsequently NLRP3 activation has also been reported to be involved in the host response to filoviruses. Incubation of THP-1 cells with inactivated Ebola virus for one hour causes NLRP3 inflammasome- and caspase-1-dependent IL-1β and IL-18 proinflammatory cytokine release. Furthermore, cytokine release is abolished upon treatment with a caspase-1 inhibitor [106]. These results show that some NLRs may play a role in the proinflammatory response of Ebola virus inside host cells; however, the role of other NLRs has yet to be elucidated in the context of filovirus infections. Furthermore, the role of NLRs during different stages of infection remains unclear. 

## 3. Innate Immune Cell Death 

The innate immune cell death resulting from PRR activation is multifaceted and can be lytic and inflammatory (pyroptosis, necroptosis, PANoptosis) as well as non-lytic and non-inflammatory (apoptosis). The cell death response is finely tuned based on which PRRs are activated and is also influenced by the cellular environment. Regulation of innate immune cell death pathways and the molecular variations known to exist in response to different stimuli have been extensively covered in other reviews [107,108,109,110]. Induction of cell death can be critical for clearing infected cells. However, inflammatory innate immune cell death can result in the release of cytokines, PAMPs, and DAMPs, and lead to a hyperinflammatory response [56]. Multiple innate immune cell death pathways have been implicated during filovirus infections.

### 3.1. Pyroptosis

Pyroptosis is a proinflammatory caspase-1–mediated lytic form of innate immune cell death [105]. In response to stimulation by PAMPs or DAMPs, caspase-1 is activated and cleaves the proinflammatory cytokines IL-1β and IL-18 as well as the pore-forming molecule GSDMD (Figure 1B) [111,112]. Caspase-1, and therefore pyroptosis, can be activated by the formation of inflammasomes, multiprotein complexes that form in response to a variety of stimuli and homeostatic perturbations. Inflammasomes include a PRR sensor, the adaptor protein apoptosis-associated speck-like protein containing a CARD (ASC), and caspase-1 [111,112]. Inflammasomes are named for their corresponding sensors, and the most well-characterized inflammasomes are NLR family pyrin domain-containing 1 (NLRP1), NLRP3, NLR family CARD-containing 4 (NLRC4), Pyrin, and absent in melanoma 2 (AIM2) [112,113,114,115,116,117,118,119,120]. In response to sensor stimulation, ASC oligomerizes into prion-like ASC specks and recruits caspase-1 to initiate pyroptotic cell death [112,121,122,123,124,125]. Release of IL-1β and IL-18 amplifies the inflammatory innate immune response [126,127,128,129,130,131]. Alternatively, inflammasomes can be activated by caspase-11 (mice) and caspase 4/5 (humans) [132]; this non-canonical inflammasome activation also releases inflammatory cytokines and causes GSDMD-mediated membrane pore formation and cell death [123,126,129,130]. Limited information is known about the relationship between pyroptosis and filovirus infection, although one study in human THP-1 cells infected with inactive Ebola virus showed that IL-1β and IL-18 are released in response to viral infection in an NLRP3 inflammasome-mediated, caspase-1-dependent manner [106]. In patients who succumbed to Ebola virus infection, IL-1β and IL-18, as well as other proinflammatory cytokines, increased during early infection and reached their highest levels approximately two days before death; the proinflammatory cytokines were up to 1000 times higher in those who succumbed to infection compared with survivors [133]. 

### 3.2. Apoptosis

Apoptosis is an important form of cell death for maintaining cellular homeostasis. It does not lyse cells or release intracellular content, but instead causes extensive membrane blebbing and shrinkage [134]. Apoptosis is initiated through either the intrinsic or extrinsic pathways. The hallmark of intrinsic apoptosis is the formation of an apoptosome, which contains apoptotic peptidase activating factor 1 (APAF1), cytochrome c, and caspase-9 (Figure 1B); the apoptosome forms in response to mitochondrial perturbations [135,136,137]. The cleavage of caspase-9, an initiator caspase, activates executioner caspases, caspase-3 and caspase-7, leading to DNA fragmentation and morphological membrane changes. Extrinsic apoptosis occurs in response to extracellular perturbations; ligand binding to apoptosis-inducing death receptors on the surface, such as Fas or TNFR1, triggers the formation of a death-inducing signaling complex that contains Fas, FADD, and the initiator caspase caspase-8 [138,139]. Caspase-8 undergoes self-cleavage upon oligomerization and then cleaves caspase-3 and caspase-7 to execute cell death [138,139]. However, caspase-8 can also induce intrinsic apoptosis through cleavage of a Bcl-2 family member, Bid, which leads to the release of cytochrome c from the mitochondria and subsequent apoptosome formation [140,141,142,143,144]. 

Lymphocyte apoptosis has long been implicated in filovirus infection, and lymphocyte and splenic apoptosis are used as markers for ebolavirus infection [145]. Analyses of cDNA microarrays from Ebola virus-infected *Macaca facicularis* (crab-eating macaques) showed an upregulation of apoptotic regulatory genes in response to infection in the early organ phase [145]. Additional studies in both nonhuman primate models and human models of Ebola virus infection found systemic intravascular apoptosis during early organ phase and late organ phase [145,146,147]. Furthermore, overexpression of the apoptotic Bcl-2 protein decreases levels of lymphocyte apoptosis, although this decrease does not correlate with an increase in survival [148]. Hepatocyte apoptosis also occurs in mice infected with a mouse-adapted strain of Ebola virus, and apoptosis is reduced in mice lacking the apoptotic proteins Bim and Bid [148]. 

### 3.3. Necroptosis

Necroptosis is a RIPK3- and MLKL-dependent form of lytic innate immune cell death executed in response to the activation of TLRs, death receptors, and IFN signaling pathways (Figure 1B) [149]. The necroptotic response to TNF-α is well documented. During TNF-α-induced necroptosis, a necrosome consisting of phosphorylated RIPK1, phosphorylated RIPK3, TRADD, and FADD forms; specifically, the physical interaction of RIPK1 and RIPK3 leads to the phosphorylation and oligomerization of MLKL, which forms pores in the membrane and induces cell death [150,151,152]. However, necroptosis is inhibited in the presence of caspase-8, which is why necroptosis is often considered a backup for other cell death pathways. Caspase-8 cleaves RIPK1, abolishing its interaction with RIPK3 to inhibit necroptosis and promote apoptosis [153]. Not much is known about necroptosis in response to filovirus infections. One recent in vitro study showed that Vero cells, HeLa cells, and primary human macrophages undergo necrotic cell death in response to Ebola virus infection [154], but whether this can be molecularly characterized as necroptosis remains unclear. A genome-wide screen of microRNAs in Ebola virus suggests that the virus encodes a microRNA that may silence host RIPK1-mediated signaling [155]. However, in-depth molecular characterization of necroptosis components is needed to understand more about whether this cell death pathway is robustly activated during filovirus infections.

### 3.4. PANoptosis

PANoptosis is a unique inflammatory innate immune cell death pathway regulated by PANoptosome complexes that integrate components from other cell death pathways [108,156,157,158,159,160,161,162]. The totality of biological effects in PANoptosis cannot be individually accounted for by pyroptosis, apoptosis, or necroptosis alone [108,157,163]. The concept of PANoptosis originated after multiple lines of biochemical, genetic, and molecular evidence highlighted the significant amount of crosstalk between molecules canonically linked to the aforementioned cell death pathways. Therefore, while PANoptosis has not been directly linked to filovirus infections to date, it has been implicated in a variety of cancers, infections, and inflammatory diseases [110], and may also play a role in filovirus infections. In some contexts, filovirus infection causes IL-1β and IL-18 release in an NLRP3 inflammasome-mediated, caspase-1-dependent manner [106]. Furthermore, inflammasome components, including NLRP3, have been identified in the formation of the PANoptosome in response to influenza A virus (IAV). To date, two PANoptosomes have been the most well-characterized, the ZBP1-PANoptosome in response to IAV or the combination of IFN and a nuclear export inhibitor [156,158,163], and the AIM2-PANoptosome in response to *Francisella novicida* and herpes simplex virus 1 (Figure 1B) [157]. It is currently unknown if filoviruses can induce the formation of PANoptosomes and, therefore, elicit PANoptosis. Furthermore, PANoptosis has been implicated in driving cytokine storm in other infections and inflammatory diseases, suggesting PANoptosis may play a role in driving filovirus-induced cytokine hyperactivation as part of a positive feedback loop between inflammatory cell death and cytokine production [56,162]. Future studies aimed at understanding filovirus infections and whether they can activate PANoptosis would provide a more comprehensive understanding of the innate immune response to these infections. 

## 4. Therapeutic Strategies and Prevention

A growing understanding of how filoviruses interact with the host innate immune system has influenced therapeutic strategies. For example, the addition of TLR agonists (poly-ICLC, MPLA, CpG 2395, allydrogel) as adjuvants to filovirus virus-like particles augments protection against Ebola virus [164]. Furthermore, the E3 ubiquitin ligase TRIM25, which promotes RIG-I recognition of viral RNA, limits filovirus replication in an IFN-dependent manner [165]. Targeting innate immune molecules involved in cytokine release and signaling has also shown preclinical promise, and inhibiting cytokine storm may be an effective strategy for many infectious and inflammatory diseases [162,166,167]. As we discover more innate immune molecules involved in filovirus pathogenesis and gain a better understanding of the molecular interplay between host and pathogen, we can begin to identify new innate immune-mediated therapeutic targets for filovirus infections. 

In addition, three filovirus-targeted vaccines, Ervebo (rVSV-EBOV), Zabdeno/Mvabea (Ad26-ZEBOV/MVA-BN-Filo), and cAd3-EBOZ [168,169,170], have shown promise in clinical trials. These vaccines express the Ebola GP, which stimulates an immune response by activating PRRs in humans [36,171]. Of these, Erbevo is the only FDA-approved vaccine for Ebola virus, and it has been effective in reducing spread in areas of outbreak [171]. Furthermore, Erbevo has been recommended for pre-exposure prophylaxis for those who are potentially at risk of contracting Ebola virus. However, the vaccines produced thus far only provide protection against Ebola virus, leaving the population unprotected against the other ebolaviruses that can infect humans [171]. In addition, many of the current vaccine strategies may not protect against emerging viral variants. More comprehensive data about the safety profiles of these new vaccines, particularly in immunocompromised and pregnant people, along with a more complete understanding of how these vaccines affect the immune system, will bring us closer to ensuring that filovirus outbreaks can be effectively managed in real time. 

Furthermore, the FDA has approved Inmazeb for the treatment of patients infected with Ebola virus. Inmazeb is a mixture of three monoclonal antibodies, atoltivimab, maftivimab, and odesivimab, which all bind separate epitopes of the Ebola virus GP. Maftivimab, a neutralizing antibody, inhibits viral entry into cells. Odesivimab, a non-neutralizing antibody, induces antibody-dependent effector functions, causing immune cell recruitment to the virus. Atoltivimab combines a neutralization mechanism with the induction of effector functions. Other GP-targeting monoclonal antibodies such as ZMapp and MR-191-N have also been shown to be effective in filovirus-infected nonhuman primates, but clinical data are limited. The PALM trial, which compared Inmazeb treatment versus ZMapp or remdesivir, found that the 28-day survival rate for patients given Inmazeb was 17% higher than those given ZMapp. However, this trial only evaluated 382 patients [172]. Additionally, any evolutionary variation in GP renders these antibodies inefficient [173]. 

Small interfering RNAs and phosphorodiamidate morpholino oligomers are also being evaluated as potential therapeutics, along with antivirals such as favipiravir and GS-5734 [174]. The use of combination therapies such as monoclonal antibodies with antiviral medications has also shown promise; in a study with Marburg virus-infected rhesus macaques, administering MR186-YTE, a Marburg-specific monoclonal antibody, and the antiviral medication remdesivir rescued animals with advanced disease [175]. Despite these advances, significant continuing work is needed to identify and implement strategies to mitigate or prevent these deadly infections. 

## 5. Limitations and Future Perspectives

Filoviruses have evolved to evade and actively counteract the host innate immune response. The result is a deadly class of viruses with a high case fatality rate. Therefore, it is critical that we have the ability to safely study these viruses to find effective therapeutics that decrease the high case fatality rate and prevent the spread of these contagious pathogens. However, working with filoviruses is difficult; they require a BSL4 containment area and intense staff training. These studies therefore require significant financial support and extensive facility preparation and maintenance. Even for those who have established BSL4 research areas, obtaining the reagents and equipment necessary to work with these deadly viruses can be challenging.

Despite these challenges, many researchers have established successful filovirus research programs using both in vitro and in vivo models with active viruses, adapted strains of viruses, as well as viral pseudotypes. Common animal models used to study filoviruses include mice, hamsters, guinea pigs, and nonhuman primates [176]. Mice are not natural hosts for many filoviruses, which leads to key differences in outcomes and lethality when comparing humans and mice. However, mice have provided important insights into pathogenesis and the efficacy of vaccine candidates and filovirus therapeutics [176]. To more closely mimic human disease, guinea pigs and hamsters can be infected with adapted strains of Ebola virus. These infections cause many clinical symptoms seen in humans, such as coagulation defects [176], and have improved the study of disease mechanisms. Additionally, nonhuman primates may be the most relevant model due to their similarities with humans; however, working with these animals is costly, requires approval from many agencies, and presents ethical challenges [176]. In lieu of working with a fully active virus, viral pseudotypes and virus-like particles are being used to study filoviruses; these require BSL2 containment and have generated informative data about how filoviruses enter cells [177]. However, this type of research is limited in its scope, as it cannot identify in vivo phenotypes and processes. 

Though many challenges exist, improved understanding of the effect of filoviruses on human hosts is critical. These viruses present a clear and present danger, as recently an outbreak of Marburg virus has been discovered in Ghana and Sudan virus has been discovered in the Republic of Uganda. Rapid isolation and contact tracing are essential to prevent the rampant spread of this deadly virus, and effective therapeutics would significantly improve patient outcomes. 

Overall, there is still much to learn about filoviruses and how they impact the human immune system. The effects of filoviruses on the innate immune system differ based on the stage of infection. A better understanding of the molecular underpinnings of how filovirus proteins interact with host innate immune components throughout the course of infection will advance our ability to create effective therapeutics, understand when to use those therapeutics, and decrease the high case fatality rate associated with this group of RNA viruses. 

## Figures and Tables

**Table 1:** Filovirus proteins and their cellular targets.

Viral Protein	Cellular Target	Outcome	References
eVP24	Karyopherin α1	Blocks nuclear translocation of phosphorylated STAT1	[89,90,91,94]
	Importin α, others	Inhibits IFN-λ1 production	[79,93]
eVP35 mVP35	Viral dsRNA	Prevents RLR-mediated recognition of virus	[84,85]
	PACT	Prevents RIG-I activation	[86,87]
	IKKε, TBK1	Decreases IRF3 phosphorylation	[88]
	IRF7	Drives sumoylation of IRF7	[88]
mVP40	JAK1, STAT1/2, TYK2	Inhibits JAK/STAT phosphorylation and nuclear translocation of STAT1/2	[92]

## Data Availability

Not applicable.
